# Angiotensin II, conventional vasopressor therapy, and mortality in shock: a large, multicenter, propensity score-weighted analysis

**DOI:** 10.1186/s13613-025-01522-3

**Published:** 2025-07-23

**Authors:** Laurence W. Busse, Caitlin ten Lohuis, Han Xu, Cooper Jannuzzo, Robert H. Lyles, J. Pedro Teixeira, Ishan Mehta, Yuan Liu

**Affiliations:** 1 Emory Critical Care Center, Emory Healthcare, Atlanta, GA USA; 2 Satellite Trials Group of Emory (STaGE), Emory University, Atlanta, GA USA; 3 Division of Pulmonary, Allergy, Critical Care and Sleep Medicine, Emory University School of Medicine, 615 Michael St, NE Ste 205, Atlanta, GA 30322 USA; 4 Department of Biostatistics and Bioinformatics, Rollins School of Public Health, Emory University, Atlanta, GA USA; 5 Office of Quality, Emory Healthcare, Atlanta, GA USA; 6 Division of Nephrology, Department of Internal Medicine, University of New Mexico School of Medicine, Albuquerque, NM USA; 7 Division of Pulmonary, Critical Care, and Sleep Medicine, Department of Internal Medicine, University of New Mexico School of Medicine, Albuquerque, NM USA

**Keywords:** Angiotensin II, Renin-angiotensin system, Shock, Vasopressors, Sepsis

## Abstract

**Background:**

Angiotensin II (Ang II) is typically used in addition to adrenergic agents and vasopressin (conventional therapy) in patients with shock, but whether its use improves outcomes is unknown.

**Research question:**

We evaluated whether Ang II, when added to conventional therapy at different norepinephrine equivalent (NE) doses, was associated with mortality.

**Methods:**

We performed a retrospective analysis of 811 patients admitted to four centers in a single healthcare system who received vasopressors for shock, including 275 who received Ang II plus conventional therapy and 536 who received only conventional therapy. Age, gender, sequential organ failure assessment score, serum lactate, background NE dose, corticosteroid use, pre-morbid angiotensin-converting enzyme inhibitor or angiotensin receptor blocker use, and Charlson Comorbidity Index were calculated at initiation of Ang II or at an equivalent point of acuity in the conventional therapy cohort. We used propensity scores with inverse probability of treatment weighting (IPTW) to achieve covariate balance and multivariable logistic regression to compare 30-day mortality, further stratifying patients by 0.10 mcg/kg/min NE increments.

**Results:**

Overall 30-day mortality was 56.4%. Groups statistically differed by all baseline variables. In multivariable logistic regression, Ang II treatment was associated with lower 30-day mortality compared to conventional therapy alone (odds ratio [OR] 0.65, 95% confidence interval [CI] 0.45–0.95, *p* = 0.025). After IPTW, Ang II use was independently associated with lower mortality (OR 0.74, 95% CI 0.55–0.99, *p* = 0.040). When stratifying by increments of background NE dose, Ang II initiation was associated with lower 30-day mortality compared to conventional therapy alone in patients on background NE doses > 0.4, > 0.5, and ≤ 0.6 mcg/kg/min. Ang II use in patients on background NE dose > 0.6 was not significantly associated with mortality.

**Conclusions:**

Ang II administration was associated with a lower risk of death in unadjusted and adjusted analyses. This effect was preserved only with patients receiving NE at doses ranging from 0.4 to 0.6 mcg/kg/min. Though additional prospective studies are required, these findings suggest that Ang II may be beneficial across a specific range of background vasopressor doses.

**Supplementary Information:**

The online version contains supplementary material available at 10.1186/s13613-025-01522-3.

## Background

Shock is a life-threatening state of acute circulatory failure with decreased tissue perfusion, inadequate cellular oxygen utilization, and organ dysfunction [1–3]. Management consists of aggressive fluid resuscitation and vasopressor support [4]. A large proportion of patients do not respond to catecholamines and vasopressin (conventional therapy). Angiotensin (Ang) II is an alternative non-catecholamine vasopressor for vasodilatory shock [5, 6]. A potent endogenous and exogenous direct vasopressor, Ang II increases mean arterial pressure via vasoconstriction and sodium reabsorption [7]. However, its optimal role is uncertain, as limited evidence exists to support the use of one vasopressor over another.

Several analyses have described the benefits of a particular vasopressor in certain shock subgroups. For example, both vasopressin and Ang II have been associated with decreased need for renal replacement therapy in patients with sepsis-associated acute kidney injury [8, 9], and Ang II has been shown to be associated with survival in patients with elevated blood renin levels [10]. Recently, attention has been paid to initiation triggers of the various vasopressor classes, with accumulating evidence suggesting benefit from early use of non-catecholamine vasopressors at lower background catecholamine doses. A post-*hoc* analysis of the Angiotensin II for the Treatment of High-Output Shock (ATHOS-3) trial reported decreased mortality in patients who received Ang II at NE doses ≤ 0.25 mcg/kg/min compared to conventional therapy, with no such effect seen at NE doses above 0.25 mcg/kg/min [11]. Similarly, a retrospective analysis of 1610 patients showed that mortality increased by 20.7% for every 10 mcg/min increase in catecholamine dose at the time of vasopressin initiation [12]. These analyses highlight the proposed benefits of multimodal vasopressor therapy [13], which are thought to stem from avoidance of the cardiotoxic and immunosuppressive effects of high-dose catecholamine therapy [14, 15].

The standard use of retrospective data to compare different vasopressor regimens pre-supposes that candidates who receive conventional therapy are comparable in severity of illness and comorbidity to those who receive Ang II. However, virtually all retrospective analyses which include patients who receive Ang II for shock describe these patients as sicker [16–18]. Propensity score methods may mitigate much of the bias due to lack of comparability or mixing of effects seen in retrospective analyses. Accordingly, using the propensity score technique of inverse probability of treatment weighting (IPTW) to achieve covariate balance, we sought to evaluate whether Ang II, when added to conventional therapy, was associated with differences in mortality in a large retrospective analysis of patients with shock. We further sought to explore whether Ang II initiation at different background NE doses was associated with risk of death.

## Methods

### Study design and data extraction

We performed a retrospective analysis of patients in a quaternary healthcare system comprised of multiple hospitals. We identified all patients who were admitted to a medical-surgical ICU and received Ang II for shock from March 2018 through August 2022, which coincided with the first 40 months that Ang II was available within our system. Ang II use in our system is used at provider discretion in patients deemed to have distributive shock, typically as a third-line agent, with initiation recommended once a patient requires catecholamines (norepinephrine dose plus epinephrine dose) at ≥0.2 mcg/kg/min as well as vasopressin (0.03–0.04 U/min). Of note, norepinephrine bitartrate is utilized in our health system and, per FDA labeling, is dosed by norepinephrine base molecule weight, not by salt weight. Patients treated with Ang II in the context of cardiopulmonary bypass were specifically excluded, as this sub-population is guided by different principles and Ang II is deployed via a different set of practice patterns [19]. We identified control patients over a similar time period who were admitted to the same medical-surgical ICUs and met the Ang II usage criteria but did not receive Ang II (catecholamines at a dose of ≥ 0.2 mcg/kg/min plus vasopressin or catecholamines at a dose of ≥ 0.3 mcg/kg/min without vasopressin). We utilized norepinephrine equivalents (NE) to describe the summative effect of catecholamines plus vasopressin in both groups, which is calculated as the sum total of norepinephrine and epinephrine, both in mcg/kg/min, + 2.5 * the dose of vasopressin (U/min). This approach has been well described and utilized in the literature [20–23]. This study was conducted in accordance with all applicable laws and local regulations, including Protection of Human Volunteers (21 CFR 50), Institutional Review Boards (21 CFR 56), and Obligations of Clinical Investigators (21 CFR 312) with a waiver of consent (IRB00113240).

All patients were identified using the medication administration record within the electronic medical record (Cerner) with data extraction from a clinical data warehouse. Once identified, patient-level data were extracted from the medical record including basic demographics, vasopressor regimen, corticosteroid use, vital signs, serum lactate levels, pre-morbid angiotensin-converting enzyme inhibitor (ACEi) / angiotensin receptor blocker (ARB) use, hemodynamic variables (when available), and 30-day mortality data (our primary outcome measure) at the time of inclusion. We identified comorbidities based on International Statistical Classification of Diseases 10th Revision (ICD-10) codes, extracted from admission data in the medical record. Sequential Organ Failure Assessment (SOFA) score and the Charlson Comorbidity Index (CCI) were calculated at the time of initiation of Ang II or at the equivalent time point in the conventional therapy cohort.

### Statistical analysis

Patients were grouped into two cohorts: those patients who received Ang II for shock (Ang II cohort) and control patients who did not receive Ang II (conventional therapy cohort). We identified, a priori, factors likely to influence a provider’s decision to use Ang II, including age, gender, SOFA score, background NE dose, CCI, and serum lactate [24–27]. However, we included all variables in our regression analyses, including concomitant corticosteroid therapy, premorbid ACEi/ARB use, and the presence/absence of high-output shock (defined below). We presented patient characteristics as descriptive statistics using mean [standard deviation] or median [interquartile range] for continuous variables and frequency (percentage) for categorical variables. We compared differences in baseline patient characteristics across cohorts via the Wilcoxon rank-sum test or two-sample t-test for continuous variables and chi-square test for categorical variables. We estimated propensity scores, defined as the conditional probability of assigning a specific treatment considering observed baseline covariates, using logistic regression. We then applied IPTW using the propensity scores and introduced a stabilized weighting method of estimating the average treatment effect (ATE_SW) to preserve sample size and reduce potential extreme weights [28]. We assessed the absolute standardized differences (ASD) before and after introducing ATE_SW into our model to ensure that the distribution of relevant covariates was similar across groups [29].

We calculated odds ratios (OR) for 30-day mortality with 95% confidence intervals (CI) comparing the two cohorts using univariate and multivariable logistic regression analysis. We further stratified each cohort by NE increments of 0.1 mcg/kg/min and performed similar analyses for strata above the cutoffs of 0.3 through 0.6 mcg/kg/min, serially enriching for a sicker population with each increment. We performed a similar analysis by analyzing stratified cohorts of patients dosed below decremental 0.1 mcg/kg/min NE cutoffs, this time enriching for a healthier population with each decrement. These strata represent subgroups of background NE dose ranges at the time of initiation of Ang II or the equivalent time point in the control arm. In our system, Ang II is recommended in patients with high-output shock features consistent with those patients included in the ATHOS-3 trial (cardiac index > 2.3 L/min/m^2^ or a combination of central or mixed venous saturation ≥70% and central venous pressure ≥8 mmHg). Despite this, neither CI nor CVP were routinely measured, and clinicians had discretion as to the determination of high- versus low-output shock. Accordingly, we performed a sensitivity analysis in patients for whom metrics of high-output shock were documented using the above criteria. All statistical tests were two-tailed with alpha set at 0.05 and all analyses were performed with SAS 9.4 (SAS Institute Inc., Cary, NC) and Winship BSR SAS macros [30].

## Results

We identified 811 patients who received vasopressors for shock, including 275 who received Ang II plus conventional therapy (Ang II group) and 536 who received only conventional therapy (conventional therapy group) (Table 1). Mean age was 62.7 years, and 45.1% (*n* = 366) were female. Mean SOFA scores, median CCI, median background NE dose, and median serum lactate were 9.7, 6, 0.41 mcg/kg/min, and 3.2 mmol/L, respectively. 67.2% (*n* = 545) of patients were treated with corticosteroids and 18.0% (*n* = 146) were on pre-morbid ACEi/ARB therapy. At baseline, patients statistically differed by all variables. Overall, 30-day mortality was 56.4%. Tables S1 and S2 show pre-IPTW univariate and multivariable logistic regression analysis for the entire cohort, as well as by NE increments. When all variables are included in the regression model for the total cohort, SOFA, serum lactate, age, and NE dose were all found to have statistically significant independent associations with mortality. Likewise, Ang II was associated with lower 30-day mortality compared to the conventional therapy only group (OR 0.65, 95% CI: 0.45–0.95, *p* = 0.025) (Table S2). Our propensity analysis is presented in Table 2. After IPTW, all variables achieved an ASD < 0.1, indicating sufficient covariate balance between groups (Supplementary Fig. 1, Panels A and B, and Table S3). In the IPTW analysis, the association between Ang II use and lower mortality was preserved (OR 0.74, 95% CI: 0.55–0.99, *p* = 0.040) (Table 2).

**Table 1:** Descriptive statistics of all variables of interest

	All Patients (*n* = 811)	Ang II (*n* = 275)	Conventional Therapy (*n* = 536)	*P*-value*
Female Gender, N (%)	366 (45.1)	109 (39.6)	257 (48.0)	0.024
Has Documented Metrics cocnistent with High-Output Shock, N (%)	126 (15.5)	60 (21.8)	66 (12.3)	0.686
Corticosteroid Use, N (%)	545 (67.2)	241 (87.6)	304 (56.7)	< 0.001
Premorbid ACEi/ARB, N (%)	146 (18.0)	84 (30.6)	62 (11.6)	< 0.001
Age, mean [SD]	62.7 [15.0]	60.5 [15.2]	63.9 [14.8]	0.002
SOFA, mean [SD]	9.7 [3.1]	11.2 [3.2]	9.0 [2.7]	< 0.001
CCI, median [IQR]	6 [4–8]	5 [3–7]	7 [4–9]	< 0.001
Lactate, median [IQR]	3.2 [1.6–6.6]	4.3 [1.9–7.9]	2.6 [1.5–5.6]	< 0.001
NE^†^, median [IQR]	0.41 [0.31–0.70]	0.48 [0.38–0.80]	0.38 [0.30–0.64]	< 0.001
30-Day Mortality	56.4%			

	Patients at NE > 0.3 (*n* = 649)	Ang II (*n* = 253)	Conventional Therapy (*n* = 396)	*P*-value*
Female Gender, N (%)	299 (46.1)	101 (39.9)	198 (50.0)	0.012
Has Documented Metrics consistent with High-Output Shock, N (%)	101 (15.6)	56 (22.1)	45 (11.4)	0.764
Corticosteroid Use, N (%)	447 (68.9)	223 (88.1)	224 (56.6)	< 0.001
Premorbid ACEi/ARB, N (%)	122 (18.8)	76 (30.0)	46 (11.6)	< 0.001
Age, mean [SD]	63.1 [15.2]	60.4 [15.2]	64.8 [14.9]	< 0.001
SOFA, mean [SD]	9.8 [3.1]	11.3 [3.2]	8.8 [2.6]	< 0.001
CCI, median [IQR]	6 [4–8]	5 [4–7]	7 [5–9]	< 0.001
Lactate, median [IQR]	3.5 [1.7–7.2]	4.3 [2.1–7.9]	2.9 [1.6–6.4]	< 0.001
NE^†^, median [IQR]	0.48 [0.38–0.80]	0.50 [0.38–0.80]	0.48 [0.37–0.80]	0.061
30-Day Mortality	57.0%			

	Patients at NE > 0.4 (*n* = 411)	Ang II (*n* = 173)	Conventional Therapy (*n* = 238)	*P*-value*
Female Gender, N (%)	182 (44.3)	62 (35.8)	120 (50.4)	0.003
Has Documented Metrics consistent with High-Output Shock, N (%)	60 (14.6)	36 (20.8)	24 (10.1)	0.353
Corticosteroid Use, N (%)	290 (70.6)	154 (89.0)	136 (57.1)	< 0.001
Premorbid ACEi/ARB, N (%)	76 (18.5)	51 (29.5)	25 (10.5)	< 0.001
Age, mean [SD]	61.8 [15.7]	59.4 [15.6]	63.5 [15.6]	0.009
SOFA, mean [SD]	9.8 [3.1]	11.3 [3.2]	8.8 [2.4]	< 0.001
CCI, median [IQR]	6 [4–8]	5 [3–7]	7 [5–9]	< 0.001
Lactate, median [IQR]	4.1 [1.9–8.2]	4.6 [2.1–8.6]	3.7 [1.7–7.8]	0.043
NE^†^, median [IQR]	0.68 [0.52–1.04]	0.68 [0.50–0.95]	0.70 [0.53–1.08]	0.364
30-Day Mortality	60.1%			

	Patients at NE > 0.5 (*n* = 310)	Ang II (*n* = 125)	Conventional Therapy (*n* = 185)	*P*-value*
Female Gender, N (%)	132 (42.6)	41 (32.8)	91 (49.2)	0.004
Has Documented Metrics consistent with High-Output Shock, N (%)	39 (12.6)	23 (18.4)	16 (8.6)	0.409
Corticosteroid Use, N (%)	220 (71.0)	113 (90.4)	107 (57.8)	< 0.001
Premorbid ACEi/ARB, N (%)	54 (17.4)	38 (30.4)	16 (8.7)	< 0.001
Age, mean [SD]	61.5 [16.2]	59.5 [16.1]	62.8 [16.1]	0.080
SOFA, mean [SD]	9.8 [3.0]	11.1 [3.3]	9.0 [2.5]	< 0.001
CCI, median [IQR]	6 [4–8]	5 [3–7]	7 [5–9]	< 0.001
Lactate, median [IQR]	5.0 [2.1–9.2]	6.0 [3.0-9.7]	4.5 [2.0-8.5]	0.046
NE^†^, median [IQR]	0.86 [0.63–1.08]	0.83 [0.63–1.10]	0.88 [0.60–1.08]	0.853
30-Day Mortality	64.8%			

	Patients at NE > 0.6 (*n* = 237)	Ang II (*n* = 100)	Conventional Therapy (*n* = 137)	*P*-value*
Female Gender, N (%)	96 (40.5)	31 (31.0)	65 (47.5)	0.011
Has Documented Metrics consistent with High-Output Shock, N (%)	27 (11.4)	15 (15.0)	12 (8.8)	0.408
Corticosteroid Use, N (%)	173 (73.0)	91 (91.0)	82 (59.9)	< 0.001
Premorbid ACEi/ARB, N (%)	42 (17.7)	29 (29.0)	13 (9.5)	< 0.001
Age, mean [SD]	61.5 [16.0]	59.9 [15.5]	62.6 [16.3]	0.199
SOFA, mean [SD]	10.1 [2.9]	11.2 [3.1]	9.3 [2.4]	< 0.001
CCI, median [IQR]	6 [4–8]	5 [3–7]	7 [5–9]	< 0.001
Lactate, median [IQR]	5.2 [2.1–9.6]	6.2 [2.7–9.8]	4.6 [2.0-9.4]	0.175
NE^†^, median [IQR]	0.97 [0.78–1.14]	0.90 [0.75–1.20]	1.00 [0.78–1.10]	0.262
30-Day Mortality	68.8%			

* The *p*-value is calculated by two-sample t-test or Wilcoxon rank sum test for continuous or ordinal covariates and chi-square test for categorical covariates. † NE in mcg/kg/min. Ang II, angiotensin II; SD, standard deviation; IQR, interquartile range; SOFA, sequential organ failure assessment; CCI, Charlson Comorbidity Index; NE, norepinephrine equivalents [norepinephrine + epinephrine + 2.5*vasopressin]; ACEi, angiotensin-converting enzyme inhibitor; ARB, angiotensin receptor blocker

**Table 2:** Mortality at different NE cutoff levels

A			Before IPTW	After IPTW
NE Cutoff	Ang II	Conventional Therapy	OR (95% CI), *P*-Value	OR (95% CI), *P*-Value
All Patients	152/275 (55.3%)	305/536 (56.9%)	**0.65 (0.45–0.95)**,** 0.025**	**0.74 (0.55–0.99)**,** 0.040**
> 0.3	141/253 (55.7%)	229/396 (57.8%)	0.74 (0.49–1.13), 0.159	0.84 (0.62–1.15), 0.285
> 0.4	97/173 (56.1%)	150/238 (63.0%)	**0.55 (0.32–0.96)**,** 0.035**	**0.64 (0.43–0.96)**,** 0.030**
> 0.5	75/125 (60.0%)	126/185 (68.1%)	**0.48 (0.26–0.91)**,** 0.023**	**0.60 (0.38–0.97)**,** 0.037**
> 0.6	66/100 (66.0%)	97/137 (70.8%)	0.72 (0.35–1.45), 0.354	0.77 (0.44–1.35), 0.363

B			Before IPTW	After IPTW
NE Cutoff	Ang II	Conventional Therapy	OR (95% CI), *P*-Value	OR (95% CI), *P*-Value
≤ 0.6	86/175 (49.1%)	208/399 (52.1%)	0.65 (0.41–1.03), 0.067	**0.68 (0.48–0.96)**,** 0.029**
≤ 0.5	77/150 (51.3%)	179/351 (51.0%)	0.85 (0.52–1.39), 0.524	0.72 (0.50–1.04), 0.080
≤ 0.4	55/102 (53.9%)	155/298 (52.0%)	0.91 (0.52–1.59), 0.733	0.72 (0.47–1.11), 0.138
≤ 0.3	11/22 (50.0%)	76/140 (54.3%)	0.58 (0.20–1.68), 0.318	0.47 (0.19–1.17), 0.106

Mortality rates of the Ang II and conventional therapy cohorts above and below various levels of NE background dose. Panel A enriches for a sicker population with each incremental NE cutoff. Specifically, the > 0.3 stratum includes all patients with a background NE dose above 0.3 mcg/kg/min at the time of Ang II initiation or the equivalent time point in the control arm, and so on. Panel B enriches for a progressively healthier population of shock patients, with each stratum including all patients for whom background NE dose was below or equal to the indicated NE cutoff, in mcg/kg/min. Specifically, the ≤ 0.6 stratum includes all patients with a background NE dose lower than or equal to 0.6 mcg/kg/min, the ≤ 0.5 stratum includes patients with background NE doses below or equal to 0.5 mcg/kg/min, and so on. Statistically significant results are in bold. For the > 0.3 stratum, the absolute standardized difference (ASD) of NE and the usage of corticosteroids were greater than 0.1 (0.123 and 0.108, respectively) after calculating the average treatment effect with stabilized weighting (ATE_SW), representing a modest imbalance in that covariate. For the > 0.4 stratum, the ASDs of CCI, lactate, NE, premorbid acei/arb administration, and the usage of corticosteroids were greater than 0.1 (0.113, 0.169, 0.162, 0.161 and 0.128, respectively) after ATE_SW. For the > 0.5 stratum, ASDs of CCI, lactate, NE and premorbid acei/arb administration were 0.131, 0.158, 0.180 and 0.158, respectively, after ATE_SW. Likewise, for the > 0.6 stratum, ASDs of NE and premorbid acei/arb administration were 0.133 and 0.186, respectively, after ATE_SW. IPTW, inverse probability of treatment weighting using propensity scores; NE, norepinephrine equivalent (norepinephrine + epinephrine + 2.5*vasopressin) dose in mcg/kg/min; OR, odds ratio; CI, confidence interval; CCI, Charlson comorbidity index; acei, angiotensin-converting enzyme inhibitor; ARB, angiotensin receptor blocker

### Stratification by NE dose

From the entire cohort, we eliminated patients at consecutive NE doses of 0.1 mcg/kg/min increments, starting with those with a background dose of NE ≤ 0.3 mcg/kg/min at the time of Ang II initiation or equivalent time point in controls, then those on NE dose ≤ 0.4 mcg/kg/min, and so forth, up to ≤ 0.6 mcg/kg/min, essentially enriching for a sicker population with each stratum. With each increment, we repeated the analysis described above. Table 2, panel A shows 30-day mortality for Ang II and conventional therapy patients above incremental background doses of NE. Both before IPTW (Table S2) and after covariates were balanced (Table 2, panel A), Ang II use was associated with lower mortality for patients when background NE doses were > 0.4 and > 0.5 mcg/kg/min. However, no mortality difference was seen when including patients with a background NE dose > 0.3 mcg/kg/min (Table 2, panel A). No mortality difference was seen when the analysis included only patients at background NE doses > 0.6 mcg/kg/min. We performed a similar analysis by eliminating patients above consecutive 0.1 mcg/kg/min cutoffs of NE to explore the effect of NE dose on mortality in a progressively healthier population (Table 2, panel B). When including all patients with background NE doses ≤ 0.6 mcg/kg/min and after IPTW, Ang II was associated with lower mortality (OR 0.68, 95% CI: 0.48–0.96, *p* = 0.029). However, for all other decrements of NE background dose ranging down to those with NE doses ≤ 0.3 mcg/kg/min, we found no independent association with mortality before or IPTW (Table 2, panel B).

We performed a sensitivity analysis by identifying all patients in each cohort that had a documented metric of cardiac output. In total, only 175 (21.5%) patients had documented metrics of cardiac output. A total of 126 (15.5%) patients had a cardiac index > 2.3 L/min/m^2^ or a combination of central or mixed venous saturation ≥70% and central venous pressure ≥8 mmHg (consistent with high-output shock), 60 of 275 (21.8%) in the Ang II group and 66 of 536 (12.3%) in the conventional therapy group. When including this as a variable in our analyses of the total cohort, the presence of documented features of high-output shock was not associated with mortality in either univariate analysis (OR 1.14, 95% CI: 0.59–2.21, *p* = 0.694) or in multivariable regression (OR 1.08, 95% CI: 0.53–2.20, *p* = 0.835). When only analyzing those patients with documented features of high-output shock, Ang II use was not associated with a mortality signal either in univariate analysis or multivariable regression, and similar results were seen when only including patients with documented features of low-output shock (*n* = 49), though the small sample sizes were significantly reduced in these analyses (Table S5). When balancing cohorts using IPTW, Ang II use was not associated with mortality in either the high-output shock sub-group or the low-output shock sub-group (though a trend toward mortality was seen in the low-output group) (Table S6).

## Discussion

In this multicenter retrospective analysis, Ang II administration was associated with a lower risk of death in a cohort of 811 patients with shock when balanced with IPTW to adjust for demographics, severity of illness, comorbidity, and other covariables. When stratifying by increasing increments in background NE doses of 0.1 mcg/kg/min to enrich for a sicker population, we found that Ang II was associated with lower mortality at NE cutoffs of > 0.4 and > 0.5 mcg/kg/min, though this effect was attenuated when including patients at NE doses > 0.3 mcg/kg/min or when only including patients > 0.6 mcg/kg/min. Ang II was also associated with lower mortality when patients receiving background doses ≤ 0.6 mcg/kg/min, though no mortality difference was seen at incrementally lower NE doses of ≤ 0.5, ≤ 0.4 and ≤ 0.3 mcg/kg/min, when enriching for a healthier population. We conclude that Ang II may mitigate the risk of death in patients and, at the very least, does not appear to confer a higher risk of mortality, at background NE doses ranging between 0.4 and 0.6 mcg/kg/min. Though our results are hypothesis-generating and additional prospective data are needed to better define the optimal use of Ang II, our results suggest that Ang II may be beneficial across certain ranges of background vasopressor doses.

Our findings build upon the current literature. Smith et al. found that Ang II had no association with 30-day mortality in a large retrospective cohort of propensity-matched patients with shock [17]. In that study, investigators matched patients by pairing each Ang II patient with one control patient from a historical period (before Ang II availability) and one concurrent patient (after Ang II availability). Our analysis utilized a different propensity score method (IPTW and ATE_SW) which avoids loss of data resulting from unmatched subjects. IPTW in conjunction with ATE_SW may be superior to propensity score matching in that it achieves a balanced level of acuity of illness by weighting while allowing for preservation of sample size and is less impacted by extreme values in the data than other methods of propensity matching. Quan et al. likewise showed no observed mortality signal with Ang II use in their study of 147 patients with shock, though the analysis was likely underpowered to do so [16]. Unlike our study, Quan et al. utilized propensity overlap weighting instead of IPTW to balance cohorts, and they did not include a measure of comorbidity as a covariable in their model. Our model includes the CCI, a validated measure of chronic illness that has been shown to contribute to morbidity and mortality in acute illness [24]. Leisman et al. evaluated 29 COVID-19 patients retrospectively, matching Ang II cases in a 1:2 ratio with controls based on date of admission, organ support, and other patient characteristics [18]. Like the study by Quan et al., this study was not designed to evaluate mortality as a primary outcome, though 6 of 10 Ang II patients died and 10 of 10 control patients died. Moreover, Leisman et al. focused exclusively on COVID-19 patients, which significantly limits generalizability. Finally, a post-*hoc* analysis by Wieruszewski of ATHOS-3 dichotomized patients by background NE dose above and below doses 0.25 mcg/kg/min and showed lower mortality with Ang II in the low-NE group compared to conventional therapy, but no difference in mortality in the high-NE group, though the ATHOS-3 trial overall reported a trend toward improved mortality [11]. This study is of limited comparability due to structural and analytical differences with ours, in that the primary endpoint of ATHOS-3 involved a blood pressure differential. Interestingly those patients classified by Wieruszewski as the high-NE group had a median baseline NE of 0.45–0.47 mcg/kg/min, which is similar to our overall patient population (median 0.41 mcg/kg/min). In contrast to results reported by Wieruszewski, we found no mortality difference when limiting our analysis to patients with NE doses ≤ 0.3 mcg/kg/min (the closest cutoff to 0.25 mcg/kg/min used in that study) (Table 2), though our data did show a trend toward lower mortality (OR 0.47, 95% CI: 0.19–1.17, *p* = 0.106). However, due to our institutional protocol for Ang II use, the number of patients initiated on Ang II at NE ≤ 0.3 mcg/kg/min was small (*n* = 22), comprising only about 8% of the Ang II cohort, limiting our ability to detect any mortality signal in this subgroup. It is possible that, were the sample size larger, our results might mirror those presented by Wieruszeweski. We believe our analysis adds upon that by Wieruszewski in that our patient population was not limited to the rather strict inclusion/exclusion criteria of the parent study (ATHOS-3), but rather included a more pragmatically obtained sample. Finally, our study is distinct in that we describe results for patients both above and below multiple background NE doses. To date, no other study has analyzed outcomes with Ang II in this way.

Measuring the comprehensive treatment effect of multiple different vasopressor combinations in shock is challenging, owing to the complex pathophysiology of shock and the immense variability of endotypes and phenotypes. This is especially true when trying to compare therapeutic options in two different observational cohorts of patients with different baseline acuity levels and different predicted outcomes. Because most providers are beholden to protocolized timing and order of deployment of vasopressors, the use of 2nd - and 3rd -line vasopressors implies a sicker patient population. We attempted to overcome this inherent bias in our retrospective analyses by utilizing IPTW based on propensity scores. One drawback of propensity matching is that it is limited by the choice of independent variables upon which to balance cohorts. We identified a priori factors that have been associated with both illness acuity but also aggressiveness of care, including age, gender, severity of acute and chronic illness (as measured by SOFA, background NE dose, and CCI), and degree of hypoperfusion (as measured by lactate). We believe that these factors may in part drive providers’ decisions to deploy 2nd - and 3rd -line vasopressors. In addition, we also included in our model factors that could plausibly influence the impact of Ang II use, including concurrent corticosteroid use, pre-morbid ACEi/ARB therapy, and the presence of documented high-output shock.

Our study has several strengths. This is one of the largest studies to date evaluating the association between Ang II and mortality, comparable to the sample size analyzed by Smith et al. [17]. Moreover, our use of stabilized IPTW with properly identified independent variables is rooted in logical physiology and well-established patterns of practice behavior. Finally, our results are in line with other published data, adding validity to our results. Our study is unique in that it is the first to present risks of mortality stratified by multiple different background NE doses.

Our study also has several limitations. Most importantly, this is a retrospective study and thus we cannot infer causality. Though we believe our model to be quite comprehensive, the possibility exists that we have not accounted for other confounders that may be driving mortality. Notable confounders absent from this analysis include serum creatinine and the need for renal replacement therapy, though assessment of renal function is partially captured within the SOFA score. Though we included pre-morbid use of ACEi or ARB therapy, were did not delineate between the two, which may have differential interactions with Ang II, and thus may contribute to our outcome of interest. Leisman et al. evaluated the MAP response of Ang II in patients on ACE inhibitor versus ARB therapy, though did not report on mortality [31]. See et al. linked ACE inhibition and ARB therapy to renin levels, and renin levels to mortality, but did not directly compare ACE inhibition versus ARB therapy on mortality in septic patients who received Ang II. To date there is no direct evidence that pre-morbid ACE inhibitor or ARB use differentially affects mortality outcomes in patients who receive Ang II for shock. Detailed hemodynamic data was only available in a small subset of our cohorts, representing a significant limitation. However, when analyzing the patients for whom we were able to find discrete measures (CI, CVP, and mixed or central venous oxygen saturation), we found that 73.3% (66 of 90) patients in the conventional therapy group and 70.5% (60 of 85) patients in the Ang II group met the criteria for high-output shock (Table S3). While we could not adjudicate the precise cause of shock in all of our patients, the similar rate of high-output shock in these two cohorts is reassuring. Moreover, the weak trend toward increased mortality seen in the low-output group, while not statistically significant, certainly reinforces the conventional knowledge regarding the use of Ang II. Additionally, our NE cutoffs are arbitrary and may not elucidate fully the relationship between NE dose and mortality with Ang II. However, our results are consistent across adjacent cutoffs, which reduces the possibility of a type I error in any one stratum. Finally, the inclusion of patients based on a therapeutic time point may introduce an immortal time or survival bias, as there were likely some patients who died before they were able to receive either Ang II or higher NE doses. However, these biases likely occurred in both cohorts.

## Conclusions

Ang II use, when added to conventional therapy, was independently associated with lower mortality in patients with shock, after controlling for baseline group imbalances using IPTW. This relationship held true for sequentially higher background NE doses of > 0.4 and > 0.5 mcg/kg/min, though the effect was extinguished at doses > 0.6 mcg/kg/min. Additionally, we observed lower mortality with the initiation of Ang II in patients with background doses < 0.6 mcg/kg/min, though not at sequentially lower levels of background NE dose. More data, including prospective trials, are needed to define the optimal role of Ang II in septic shock, including those that focus on patients with AKI or needing RRT, those on RAAS inhibition therapy, and in those with mixed shock. However, these results suggest that Ang II initiation may be beneficial across a range of background vasopressor doses of 0.4 to 0.6 mcg/kg/min of NE, and that clinicians may consider the initiation of Ang II in selected patients with shock unresponsive to moderate-to-high doses of conventional vasopressor therapy.

## Electronic supplementary material

Below is the link to the electronic supplementary material.

Supplementary Material 1

Supplementary Material 2

Supplementary Material 3

Supplementary Material 4

Supplementary Material 5

## Data Availability

The datasets used and/or analyzed during the current study are available from the corresponding author on reasonable request.

## Abbreviations

ACEi: Angiotensin-converting enzyme inhibitor
Ang II: Angiotensin II
ARB: Angiotensin receptor blocker
ASD: Absolute standardized difference
ATE_SW: Stabilized weighting method of estimating the average treatment effect
ATHOS-3: Angiotensin II for the Treatment of High-Output Shock trial
CCI: Charlson Comorbidity Index
CI: Confidence interval
ICD-10: International Statistical Classification of Diseases − 10th Revision
IPTW: Inverse probability treatment weighting
NE: Norepinephrine equivalent
OR: Odds ratio
SOFA: Sequential organ failure assessment
