# Supplementary material for: Angiotensin II, conventional vasopressor therapy, and mortality in shock: a large, multicenter, propensity score-weighted analysis
Source: Ann Intensive Care. 2025 Jul 23;15:104. doi: 10.1186/s13613-025-01522-3 (PMC12286902; doi:10.1186/s13613-025-01522-3)
Supplement: Supplementary file 1 — Supplementary Material 1 [file 13613_2025_1522_MOESM1_ESM.docx]

**Figure S1: Propensity balancing**


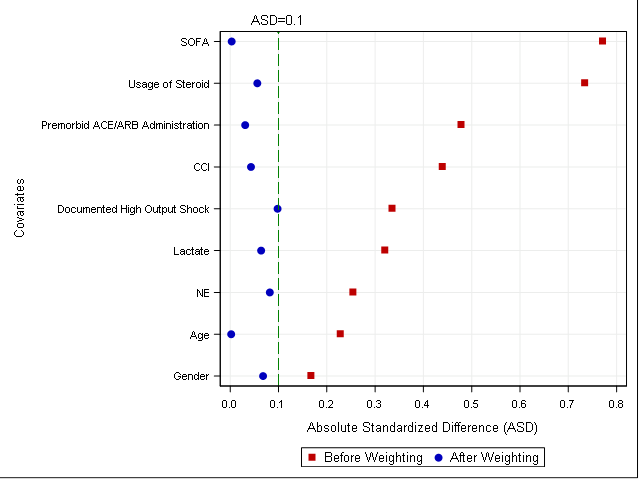

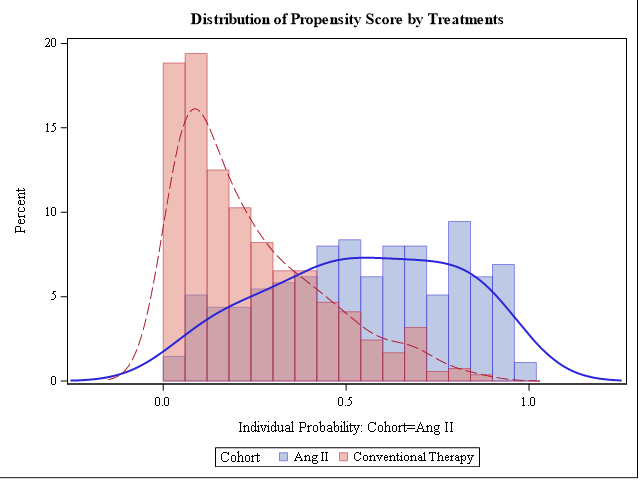
A B

Legend:

Panel A: Distribution of propensity score by treatment assignment among all 811 patients. Panel B: Absolute standardized difference before and after **PS ATE_SW** adjustment among all 811 patients. Ang II, angiotensin II; SOFA, sequential organ failure assessment; ACE, angiotensin converting enzyme inhibitor; ARB, angiotensin receptor blocker; CCI, Charlson Comorbidity Index; PS, propensity score; ATE_SW, stabilized weighting in the estimation of average treatment effect; NE, norepinephrine equivalents (norepinephrine + epinephrine + 2.5*Vasopressin).
