# Supplementary material for: Angiotensin II, conventional vasopressor therapy, and mortality in shock: a large, multicenter, propensity score-weighted analysis
Source: Ann Intensive Care. 2025 Jul 23;15:104. doi: 10.1186/s13613-025-01522-3 (PMC12286902; doi:10.1186/s13613-025-01522-3)
Supplement: Supplementary file 2 — Supplementary Material 2 [file 13613_2025_1522_MOESM2_ESM.docx]

**Table S1: Univariate Analysis of 30-day Mortality**

|  | **Died - Entire Cohort** | | **Univariate Analysis** | |
| --- | --- | --- | --- | --- |
|  | **No (n=354)** | **Yes (n=457)** | **Odds Ratio (95% CI)** | **P-value** |
| Female (n=366) | 167 (47.2%) | 199 (43.5%) | 0.86 (0.65-1.14) | 0.303 |
| Documented High-Output Shock (n=126) | 55 (15.5) | 71 (15.5) | 1.14 (0.59-2.21) | 0.694 |
| Corticosteroid Use (n=545) | 237 (67.0) | 308 (67.4) | 1.02 (0.76-1.37) | 0.893 |
| Premorbid ACEi/ARB (n=146) | 70 (19.8) | 76 (16.6) | 0.81 (0.57-1.16) | 0.248 |
| SOFA, mean (SD) | 9.2 (3.1) | 10.2 (3.0) | 1.11 (1.06-1.16) | <0.001 |
| CCI, median (IQR) | 6 (4-8) | 6 (4-9) | 1.09 (1.04-1.14) | <0.001 |
| Lactate, median (IQR) | 2.2 (1.3-4.3) | 4.0 (1.9-7.8) | 1.13 (1.08-1.17) | <0.001 |
| Age, mean (SD) | 60.4 (15.4) | 64.3 (14.5) | 1.02 (1.01-1.03) | <0.001 |
| NE, median (IQR) | 0.40 (0.30-0.58) | 0.45 (0.32-0.80) | 2.35 (1.61-3.44) | <0.001 |
| Ang II, n (%) | 123 (34.8%) | 152 (33.3%) | 0.94 (0.70-1.25) | 0.657 |
|  |  |  |  |  |
|  | **Died - NE > 0.3** | | **Univariate Analysis** | |
|  | **No (n=279)** | **Yes (n=370)** | **Odds Ratio (95% CI)** | **P-value** |
| Female (n=299) | 138 (49.5%) | 161 (43.5%) | 0.79 (0.58-1.08) | 0.133 |
| Documented High-Output Shock (n=101) | 45 (16.1) | 56 (15.1) | 0.99 (0.46-2.14) | 0.991 |
| Corticosteroid Use (n=447) | 193 (69.2) | 254 (68.7) | 0.98 (0.70-1.37) | 0.886 |
| Premorbid ACEi/ARB (n=122) | 59 (21.2) | 63 (17.0) | 0.77 (0.52-1.14) | 0.184 |
| SOFA, mean (SD) | 9.4 (3.1) | 10.1 (3.0) | 1.09 (1.03-1.15) | 0.001 |
| CCI, median (IQR) | 6 (4-8) | 6 (4-8) | 1.08 (1.02-1.14) | 0.006 |
| Lactate, median (IQR) | 2.5 (1.4-5.3) | 4.5 (2.0-8.6) | 1.12 (1.01-1.03) | <0.001 |
| Age, mean (SD) | 61.1 (15.5) | 64.5 (14.8) | 1.01 (1.00-1.03) | 0.005 |
| NE, median (IQR) | 0.45 (0.37-0.63) | 0.58 (0.38-0.95) | 2.57 (1.69-3.90) | <0.001 |
| Ang II, n (%) | 112 (40.1%) | 141 (38.1%) | 0.92 (0.67-1.26) | 0.598 |
|  |  |  |  |  |
|  | **Died - NE > 0.4** | | **Univariate Analysis** | |
|  | **No (n=164)** | **Yes (n=247)** | **Odds Ratio (95% CI)** | **P-value** |
| Female (n=182) | 80 (48.8%) | 102 (41.3%) | 0.74 (0.50-1.10) | 0.135 |
| Documented High-Output Shock (n=60) | 28 (17.1) | 32 (13.0) | 0.69 (0.26-1.81) | 0.446 |
| Corticosteroid Use (n=290) | 117 (71.3) | 173 (70.0) | 0.94 (0.61-1.45) | 0.777 |
| Premorbid ACEi/ARB (n=76) | 33 (20.1) | 43 (17.4) | 0.84 (0.51-1.38) | 0.488 |
| SOFA, mean (SD) | 9.5 (3.2) | 10.1 (2.9) | 1.07 (1.00-1.14) | 0.047 |
| CCI, median (IQR) | 6 (4-8) | 6 (4-8) | 1.06 (0.99-1.14) | 0.073 |
| Lactate, median (IQR) | 3.1 (1.4-6.1) | 5.2 (2.3-9.8) | 1.13 (1.07-1.19) | <0.001 |
| Age, mean (SD) | 59.5 (16.2) | 63.3 (15.2) | 1.02 (1.00-1.03) | 0.017 |
| NE, median (IQR) | 0.58 (0.48-0.88) | 0.78 (0.58-1.08) | 2.91 (1.73-4.90) | <0.001 |
| Ang II, n (%) | 76 (46.3) | 97 (39.3) | 0.75 (0.50-1.12) | 0.156 |
|  |  |  |  |  |
|  | **Died - NE > 0.5** | | **Univariate Analysis** | |
|  | **No (n=109)** | **Yes (n=201)** | **Odds Ratio (95% CI)** | **P-value** |
| Female (n=132) | 48 (44.0%) | 84 (41.8%) | 0.91 (0.57-1.46) | 0.703 |
| Documented High-Output Shock (n=39) | 15 (13.8) | 24 (11.9) | 0.87 (0.27-2.86) | 0.822 |
| Corticosteroid Use (n=220) | 78 (71.6) | 142 (70.7) | 0.96 (0.57-1.60) | 0.866 |
| Premorbid ACEi/ARB (n=54) | 20 (18.4) | 34 (16.9) | 0.91 (0.49-1.67) | 0.751 |
| SOFA, mean (SD) | 9.4 (3.2) | 10.1 (2.9) | 1.08 (0.99-1.16) | 0.075 |
| CCI, median (IQR) | 5 (4-8) | 6 (4-8) | 1.06 (0.98-1.14) | 0.158 |
| Lactate, median (IQR) | 3.6 (1.6-7.0) | 6.1 (2.6-10.2) | 1.11 (1.05-1.17) | <0.001 |
| Age, mean (SD) | 58.3 (16.7) | 63.2 (15.6) | 1.02 (1.00-1.03) | 0.012 |
| NE, median (IQR) | 0.74 (0.59-0.98) | 0.89 (0.68-1.10) | 2.38 (1.34-4.21) | 0.003 |
| Ang II, n (%) | 50 (45.9%) | 75 (37.3%) | 0.82 (0.47-1.44) | 0.496 |
|  |  |  |  |  |
|  | **Died - NE > 0.6** | | **Univariate Analysis** | |
|  | **No (n=74)** | **Yes (n=163)** | **Odds Ratio (95% CI)** | **P-value** |
| Female (n=96) | 30 (40.5%) | 66 (40.5%) | 0.99 (0.57-1.75) | 0.994 |
| Documented High-Output Shock (n=27) | 9 (12.2) | 18 (11.0) | 0.60 (0.13-2.74) | 0.510 |
| Corticosteroid Use (n=173) | 55 (74.3) | 118 (72.4) | 0.91 (0.49-1.69) | 0.756 |
| Premorbid ACEi/ARB (n=42) | 14 (18.9) | 28 (17.2) | 0.89 (0.44-1.81) | 0.745 |
| SOFA, mean (SD) | 9.9 (3.1) | 10.2 (2.8) | 1.03 (0.94-1.14) | 0.499 |
| CCI, median (IQR) | 5 (4-8) | 6 (4-8) | 1.06 (0.97-1.17) | 0.180 |
| Lactate, median (IQR) | 3.3 (1.4-8.1) | 6.0 (2.7-10.4) | 1.10 (1.03-1.17) | 0.004 |
| Age, mean (SD) | 57.5 (17.3) | 63.4 (14.2) | 1.02 (1.00-1.04) | 0.020 |
| NE, median (IQR) | 0.90 (0.73-1.10) | 1.00 (0.78-1.28) | 2.01 (1.07-3.78) | 0.030 |
| Ang II, n (%) | 34 (46.0%) | 66 (40.5%) | 0.80 (0.46-1.39) | 0.431 |

Table S1 Legend: Characteristics associated with 30-day mortality in the entire cohort, and with each progressively increasing NE stratum, in dose increments of 0.1 mcg/kg/min. The univariate analysis shows the odds ratio of mortality for each covariate. CI, confidence interval; ACEi, angiotensin converting enzyme inhibitor; ARB, angiotensin receptor blocker; SOFA, sequential organ failure assessment; CCI, Charlson Comorbidity Index; NE, norepinephrine equivalents (norepinephrine + epinephrine + 2.5*vasopressin). Ang II, angiotensin II.
