# Supplementary material for: Angiotensin II, conventional vasopressor therapy, and mortality in shock: a large, multicenter, propensity score-weighted analysis
Source: Ann Intensive Care. 2025 Jul 23;15:104. doi: 10.1186/s13613-025-01522-3 (PMC12286902; doi:10.1186/s13613-025-01522-3)
Supplement: Supplementary file 3 — Supplementary Material 3 [file 13613_2025_1522_MOESM3_ESM.docx]

**Table S2: Multivariable Regression of 30-day Mortality**

|  | **Died - Entire Cohort** | | **Multivariable Regression** | | |
| --- | --- | --- | --- | --- | --- |
|  | **No (n=354)** | **Yes (n=457)** | **Odds Ratio (95% CI)** | | **P-value** |
| Female (n=366) | 167 (47.2%) | 199 (43.5%) | 0.88 (0.65-1.19) | | 0.418 |
| Documented High-Output Shock (n=126) | 55 (15.5) | 71 (15.5) | 1.08 (0.53-2.20) | | 0.835 |
| Corticosteroid Use (n=545) | 237 (67.0) | 308 (67.4) | 1.00 (0.72-1.40) | | 0.987 |
| Premorbid ACEi/ARB (n=146) | 70 (19.8) | 76 (16.6) | 0.74 (0.49-1.10) | | 0.131 |
| SOFA, mean (SD) | 9.2 (3.1) | 10.2 (3.0) | 1.14 (1.08-1.20) | | <0.001 |
| CCI, median (IQR) | 6 (4-8) | 6 (4-9) | 1.04 (0.98-1.10) | | 0.180 |
| Lactate, median (IQR) | 2.2 (1.3-4.3) | 4.0 (1.9-7.8) | 1.12 (1.07-1.16) | | <0.001 |
| Age, mean (SD) | 60.4 (15.4) | 64.3 (14.5) | 1.02 (1.01-1.03) | | <0.001 |
| NE, median (IQR) | 0.40 (0.30-0.58) | 0.45 (0.32-0.80) | 2.10 (1.42-3.10) | | <0.001 |
| Ang II, n (%) | 123 (34.8%) | 152 (33.3%) | 0.65 (0.45-0.95) | | 0.025 |
|  |  |  |  | |  |
|  | **Died - NE > 0.3** | | **Multivariable Regression** | | |
|  | **No (n=279)** | **Yes (n=370)** | **Odds Ratio (95% CI)** | | **P-value** |
| Female (n=299) | 138 (49.5%) | 161 (43.5%) | 0.80 (0.57-1.12) | | 0.197 |
| Documented High-Output Shock (n=101) | 45 (16.1) | 56 (15.1) | 1.03 (0.45-2.34) | | 0.950 |
| Corticosteroid Use (n=447) | 193 (69.2) | 254 (68.7) | 0.96 (0.65-1.41) | | 0.831 |
| Premorbid ACEi/ARB (n=122) | 59 (21.2) | 63 (17.0) | 0.69 (0.44-1.07) | | 0.093 |
| SOFA, mean (SD) | 9.4 (3.1) | 10.1 (3.0) | 1.12 (1.05-1.19) | | <0.001 |
| CCI, median (IQR) | 6 (4-8) | 6 (4-8) | 1.04 (0.97-1.11) | | 0.247 |
| Lactate, median (IQR) | 2.5 (1.4-5.3) | 4.5 (2.0-8.6) | 1.12 (1.07-1.16) | | <0.001 |
| Age, mean (SD) | 61.1 (15.5) | 64.5 (14.8) | 1.02 (1.01-1.04) | | 0.001 |
| NE, median (IQR) | 0.45 (0.37-0.63) | 0.58 (0.38-0.95) | 2.34 (1.52-3.59) | | <0.001 |
| Ang II, n (%) | 112 (40.1%) | 141 (38.1%) | 0.74 (0.49-1.13) | | 0.159 |
|  |  |  |  | |  |
|  | **Died - NE > 0.4** | | **Multivariable Regression** | | |
|  | **No (n=164)** | **Yes (n=247)** | **Odds Ratio (95% CI)** | **P-value** | |
| Female (n=182) | 80 (48.8%) | 102 (41.3%) | 0.69 (0.44-1.07) | 0.100 | |
| Documented High-Output Shock (n=60) | 28 (17.1) | 32 (13.0) | 0.68 (0.24-1.93) | 0.468 | |
| Corticosteroid Use (n=290) | 117 (71.3) | 173 (70.0) | 1.09 (0.65-1.81) | 0.751 | |
| Premorbid ACEi/ARB (n=76) | 33 (20.1) | 43 (17.4) | 0.76 (0.43-1.35) | 0.347 | |
| SOFA, mean (SD) | 9.5 (3.2) | 10.1 (2.9) | 1.11 (1.02-1.20) | 0.015 | |
| CCI, median (IQR) | 6 (4-8) | 6 (4-8) | 1.01 (0.93-1.10) | 0.799 | |
| Lactate, median (IQR) | 3.1 (1.4-6.1) | 5.2 (2.3-9.8) | 1.13 (1.07-1.19) | <0.001 | |
| Age, mean (SD) | 59.5 (16.2) | 63.3 (15.2) | 1.02 (1.01-1.04) | 0.011 | |
| NE, median (IQR) | 0.58 (0.48-0.88) | 0.78 (0.58-1.08) | 2.41 (1.44-4.03) | <0.001 | |
| Ang II, n (%) | 76 (46.3) | 97 (39.3) | 0.55 (0.32-0.96) | 0.035 | |
|  |  |  |  |  | |
|  | **Died - NE > 0.5** | | **Multivariable Regression** | | |
|  | **No (n=109)** | **Yes (n=201)** | **Odds Ratio (95% CI)** | **P-value** | |
| Female (n=132) | 48 (44.0%) | 84 (41.8%) | 0.82 (0.48-1.39) | 0.452 | |
| Documented High-Output Shock (n=39) | 15 (13.8) | 24 (11.9) | 0.84 (0.23-3.06) | 0.792 | |
| Corticosteroid Use (n=220) | 78 (71.6) | 142 (70.7) | 1.15 (0.62-2.11) | 0.663 | |
| Premorbid ACEi/ARB (n=54) | 20 (18.4) | 34 (16.9) | 0.77 (0.39-1.54) | 0.465 | |
| SOFA, mean (SD) | 9.4 (3.2) | 10.1 (2.9) | 1.11 (1.01-1.23) | 0.030 | |
| CCI, median (IQR) | 5 (4-8) | 6 (4-8) | 0.98 (0.89-1.08) | 0.673 | |
| Lactate, median (IQR) | 3.6 (1.6-7.0) | 6.1 (2.6-10.2) | 1.12 (1.05-1.19) | <0.001 | |
| Age, mean (SD) | 58.3 (16.7) | 63.2 (15.6) | 1.03 (1.01-1.05) | 0.006 | |
| NE, median (IQR) | 0.74 (0.59-0.98) | 0.89 (0.68-1.10) | 2.28 (1.28-4.07) | 0.005 | |
| Ang II, n (%) | 50 (45.9%) | 75 (37.3%) | 0.48 (0.26-0.91) | 0.023 | |
|  |  |  |  |  | |
|  | **Died - NE > 0.6** | | **Multivariable Regression** | | |
|  | **No (n=74)** | **Yes (n=163)** | **Odds Ratio (95% CI)** | **P-value** | |
| Female (n=96) | 30 (40.5%) | 66 (40.5%) | 0.87 (0.47-1.61) | 0.653 | |
| Documented High-Output Shock (n=27) | 9 (12.2) | 18 (11.0) | 0.58 (0.12-2.92) | 0.512 | |
| Corticosteroid Use (n=173) | 55 (74.3) | 118 (72.4) | 1.01 (0.49-2.10) | 0.973 | |
| Premorbid ACEi/ARB (n=42) | 14 (18.9) | 28 (17.2) | 0.68 (0.31-1.49) | 0.329 | |
| SOFA, mean (SD) | 9.9 (3.1) | 10.2 (2.8) | 1.06 (0.94-1.19) | 0.343 | |
| CCI, median (IQR) | 5 (4-8) | 6 (4-8) | 1.01 (0.90-1.13) | 0.907 | |
| Lactate, median (IQR) | 3.3 (1.4-8.1) | 6.0 (2.7-10.4) | 1.10 (1.03-1.17) | 0.005 | |
| Age, mean (SD) | 57.5 (17.3) | 63.4 (14.2) | 1.02 (1.00-1.05) | 0.031 | |
| NE, median (IQR) | 0.90 (0.73-1.10) | 1.00 (0.78-1.28) | 2.12 (1.10-4.07) | 0.024 | |
| Ang II, n (%) | 34 (46.0%) | 66 (40.5%) | 0.72 (0.35-1.45) | 0.354 | |

Table S2 Legend: Characteristics associated with 30-day mortality in the entire cohort, and with each progressively increasing NE stratum, in dose increments of 0.1 mcg/kg/min. The regression analysis includes all variables in the model. CI, confidence interval; ACEi, angiotensin converting enzyme inhibitor; ARB, angiotensin receptor blocker; SOFA, sequential organ failure assessment; CCI, Charlson Comorbidity Index; NE, norepinephrine equivalents (norepinephrine + epinephrine + 2.5*vasopressin). Ang II, angiotensin II.
