# Supplementary material for: Angiotensin II, conventional vasopressor therapy, and mortality in shock: a large, multicenter, propensity score-weighted analysis
Source: Ann Intensive Care. 2025 Jul 23;15:104. doi: 10.1186/s13613-025-01522-3 (PMC12286902; doi:10.1186/s13613-025-01522-3)
Supplement: Supplementary file 4 — Supplementary Material 4 [file 13613_2025_1522_MOESM4_ESM.docx]

Table S3: Absolute Standardized Difference of baseline covariates before and after inverse probability of treatment weighting using propensity scores among all 811 patients

|  | | **Study Sample Distribution** | | **Absolute Standardized**  **Difference (ASD)** | |
| --- | --- | --- | --- | --- | --- |
|  | | **____________________________** | | **____________________________** | |
| **Covariate** | **Level** | **Before** | **After** | **Before** | **After** |
| SOFA | Mean (SD) | 9.7 (3.1) | 9.5 (2.9) | **0.772** | 0.003 |
|  | | | | | |
| CCI | Mean (SD) | 6.3 (3) | 6.3 (3) | **0.44** | 0.043 |
|  | | | | | |
| Lactate | Mean (SD) | 4.7 (4.3) | 4.8 (4.7) | **0.321** | 0.064 |
|  | | | | | |
| Age | Mean (SD) | 62.7 (15) | 63.1 (14.8) | **0.229** | 0.002 |
|  | | | | | |
| NE | Mean (SD) | 0.6 (0.5) | 0.6 (0.4) | **0.255** | 0.082 |
|  | | | | | |
| Sex | F | 366 (45.1) | 382 (47.4) | **0.168** | 0.068 |
|  | M | 445 (54.9) | 425 (52.6) | **0.168** | 0.068 |
|  | | | | | |
| Documented High-Output Shock | No | 49 (6) | 60 (7.4) | **0.184** | 0.098 |
|  | Yes | 126 (15.5) | 108 (13.4) | **0.255** | 0.048 |
|  | | | | | |
| Premorbid ACEi/ARB Administration | No | 665 (82) | 676 (83.8) | **0.479** | 0.031 |
|  | Yes | 146 (18) | 131 (16.2) | **0.479** | 0.031 |
|  | | | | | |
| Corticosteroid Use | No | 266 (32.8) | 280 (34.7) | **0.735** | 0.056 |
|  | Yes | 545 (67.2) | 527 (65.3) | **0.735** | 0.056 |
| * Absolute standardized differences (ASD) ≥0.1 are in bold and indicate significant imbalance.  * * SD, standard deviation; SOFA, sequential organ failure assessment; CCI, Charlson Comorbidity Index; NE, norepinephrine equivalents (norepinephrine + epinephrine + 2.5*vasopressin); ACEi, angiotensin converting enzyme inhibitor; ARB, angiotensin receptor blocker. | | | | | |
