# Supplementary material for: Angiotensin II, conventional vasopressor therapy, and mortality in shock: a large, multicenter, propensity score-weighted analysis
Source: Ann Intensive Care. 2025 Jul 23;15:104. doi: 10.1186/s13613-025-01522-3 (PMC12286902; doi:10.1186/s13613-025-01522-3)
Supplement: Supplementary file 5 — Supplementary Material 5 [file 13613_2025_1522_MOESM5_ESM.docx]

**Table S4: Descriptive statistics of all variables of interest in patients with documented shock metrics**

|  | **All Patients (n=811)** | **Ang II (n=275)** | **Conventional Therapy (n=536)** | **P-value*** |
| --- | --- | --- | --- | --- |
| Female Gender, N (%) | 366 (45.1) | 109 (39.6) | 257 (48.0) | 0.024 |
| Documented High Output Shock, N (%) | 126 (15.5) | 60 (21.8) | 66 (12.3) | 0.686 |
| Steroid Use, N (%) | 545 (67.2) | 241 (87.6) | 304 (56.7) | <0.001 |
| Premorbid ACE/ARB, N (%) | 146 (18.0) | 84 (30.6) | 62 (11.6) | <0.001 |
| Age, mean [SD] | 62.7 [15.0] | 60.5 [15.2] | 63.9 [14.8] | 0.002 |
| SOFA, mean [SD] | 9.7 [3.1] | 11.2 [3.2] | 9.0 [2.7] | <0.001 |
| CCI, median [IQR] | 6 [4-8] | 5 [3-7] | 7 [4-9] | <0.001 |
| Lactate, median [IQR] | 3.2 [1.6-6.6] | 4.3 [1.9-7.9] | 2.6 [1.5-5.6] | <0.001 |
| NE^†^, median [IQR] | 0.41 [0.31-0.70] | 0.48 [0.38-0.80] | 0.38 [0.30-0.64] | <0.001 |
| 30-Day Mortality | 56.4% |  |  |  |
|  |  |  |  |  |
|  | **High Output Shock (n=126)** | **Ang II (n=60)** | **Conventional Therapy (n=66)** | **P-value*** |
| Female Gender, N (%) | 45 (35.7) | 23 (38.3) | 22 (33.3) | 0.559 |
| Steroid Use, N (%) | 101 (80.2) | 60 (100.0) | 41 (62.1) | <0.001 |
| Premorbid ACE/ARB, N (%) | 23 (18.3) | 13 (21.7) | 10 (15.2) | 0.344 |
| Age, mean [SD] | 57.6 [14.2] | 55.0 [15.3] | 60.0 [12.8] | 0.049 |
| SOFA, mean [SD] | 11.2 [3.1] | 12.7 [2.9] | 10.0 [2.8] | <0.001 |
| CCI, median [IQR] | 6 [4-9] | 5 [3-7] | 7 [5-9] | <0.001 |
| Lactate, median [IQR] | 4.2 [2.0-6.7] | 4.6 [3.7-7.5] | 3.6 [1.7-6.0] | 0.018 |
| NE^†^, median [IQR] | 0.40 [0.32-0.58] | 0.44 [0.36-0.60] | 0.34 [0.28-0.50] | 0.002 |
| 30-Day Mortality | 56.4% |  |  |  |
|  |  |  |  |  |
|  | **Low Output Shock (n=49)** | **Ang II (n=25)** | **Conventional Therapy (n=24)** | **P-value*** |
| Female Gender, N (%) | 21 (42.9) | 5 (20.0) | 16 (66.7) | 0.001 |
| Steroid Use, N (%) | 37 (75.5) | 20 (80.0) | 17 (70.8) | 0.456 |
| Premorbid ACE/ARB, N (%) | 14 (28.6) | 10 (40.0) | 4 (16.7) | 0.114 |
| Age, mean [SD] | 67.0 [12.2] | 65.1 [13.0] | 69.0 [11.3] | 0.266 |
| SOFA, mean [SD] | 9.7 [2.6] | 10.5 [2.9] | 8.9 [2.0] | 0.028 |
| CCI, median [IQR] | 6 [3-8] | 5 [3-7] | 6 [3-9] | 0.622 |
| Lactate, median [IQR] | 3.7 [1.9-6.7] | 4.1 [1.8-8.9] | 3.1 [2.0-5.0] | 0.407 |
| NE^†^, median [IQR] | 0.40 [0.29-0.78] | 0.48 [0.38-0.83] | 0.36 [0.28-0.44] | 0.013 |
| 30-Day Mortality | 53.1% |  |  |  |
|  |  |  |  |  |

* The p-value is calculated by two-sample t-test or Wilcoxon rank sum test for numerical covariates and chi-square test for categorical covariates. † NE is dosed in mcg/kg/min. Ang II, angiotensin II; ACE, angiotensin converting enzyme inhibitor therapy; ARB, angiotensin receptor blocker therapy; SD, standard deviation; IQR, interquartile range; SOFA, sequential organ failure assessment; CCI, Charlson Comorbidity Index; NE, norepinephrine equivalents [norepinephrine + epinephrine + 2.5*vasopressin].

**Table S5: Univariate Analysis and Multivariable Regression of 30-day Mortality**

|  | **Died - Entire Cohort** | | **Univariate Analysis** | | | **Multivariable Regression** | | |
| --- | --- | --- | --- | --- | --- | --- | --- | --- |
|  | **No**  **(n=354)** | **Yes**  **(n=457)** | **Odds Ratio (95% CI)** | | **P-value** | **Odds Ratio (95% CI)** | | **P-value** |
| Female (n=366) | 167 (47.2%) | 199 (43.5%) | 0.86 (0.65-1.14) | | 0.303 | 0.88 (0.65-1.19) | | 0.418 |
| Documented High Output Shock (n=126) | 55 (15.5) | 71 (15.5) | 1.14 (0.59-2.21) | | 0.694 | 1.08 (0.53-2.20) | | 0.835 |
| Steroid Use (n=545) | 237 (67.0) | 308 (67.4) | 1.02 (0.76-1.37) | | 0.893 | 1.00 (0.72-1.40) | | 0.987 |
| Premorbid ACE/ARB (n=146) | 70 (19.8) | 76 (16.6) | 0.81 (0.57-1.16) | | 0.248 | 0.74 (0.49-1.10) | | 0.131 |
| SOFA, mean (SD) | 9.2 (3.1) | 10.2 (3.0) | 1.11 (1.06-1.16) | | <0.001 | 1.14 (1.08-1.20) | | <0.001 |
| CCI, median (IQR) | 6 (4-8) | 6 (4-9) | 1.09 (1.04-1.14) | | <0.001 | 1.04 (0.98-1.10) | | 0.180 |
| Lactate, median (IQR) | 2.2 (1.3-4.3) | 4.0 (1.9-7.8) | 1.13 (1.08-1.17) | | <0.001 | 1.12 (1.07-1.16) | | <0.001 |
| Age, mean (SD) | 60.4 (15.4) | 64.3 (14.5) | 1.02 (1.01-1.03) | | <0.001 | 1.02 (1.01-1.03) | | <0.001 |
| NE, median (IQR) | 0.40 (0.30-0.58) | 0.45 (0.32-0.80) | 2.35 (1.61-3.44) | | <0.001 | 2.10 (1.42-3.10) | | <0.001 |
| Ang II, n (%) | 123 (34.8%) | 152 (33.3%) | 0.94 (0.70-1.25) | | 0.657 | 0.65 (0.45-0.95) | | 0.025 |
|  |  |  |  | |  |  | |  |
|  | **Died – High Output Shock** | | **Univariate Analysis** | | | **Multivariable Regression** | | |
|  | **No**  **(n=55)** | **Yes**  **(n=71)** | **Odds Ratio (95% CI)** | | **P-value** | **Odds Ratio (95% CI)** | | **P-value** |
| Female (n=45) | 18 (32.7%) | 27 (38.0%) | 1.26 (0.60-2.64) | | 0.538 | 1.73 (0.72-4.12) | | 0.218 |
| Steroid Use (n=101) | 49 (89.1) | 52 (73.2) | 0.34 (0.12-0.91) | | 0.032 | 0.29 (0.09-0.96) | | 0.042 |
| Premorbid ACE/ARB (n=23) | 11 (20.0) | 12 (16.9) | 0.81 (0.33-2.01) | | 0.656 | 0.86 (0.29-2.56) | | 0.781 |
| SOFA, mean (SD) | 10.3 (3.3) | 12.0 (2.8) | 1.20 (1.06-1.35) | | 0.004 | 1.26 (1.08-1.47) | | 0.003 |
| CCI, median (IQR) | 6 (4-9) | 6 (4-8) | 0.99 (0.89-1.12) | | 0.987 | 0.97 (0.83-1.13) | | 0.717 |
| Lactate, median (IQR) | 3.6 (1.3-5.3) | 5.3 (3.5-7.9) | 1.16 (1.04-1.29) | | 0.006 | 1.14 (1.02-1.28) | | 0.022 |
| Age, mean (SD) | 57.5 (14.0) | 57.7 (14.4) | 1.00 (0.98-1.03) | | 0.944 | 1.03 (0.99-1.07) | | 0.153 |
| NE, median (IQR) | 0.43 (0.32-0.53) | 0.40 (0.31-0.63) | 1.37 (0.49-3.83) | | 0.553 | 1.27 (0.36-4.47) | | 0.712 |
| Ang II, n (%) | 25 (45.5%) | 35 (49.3%) | 1.17 (0.58-2.36) | | 0.669 | 0.87 (0.32-2.37) | | 0.784 |
|  |  |  |  | |  |  | |  |
|  | **Died – Low Output Shock** | | **Univariate Analysis** | | | **Multivariable Regression** | | |
|  | **No**  **(n=23)** | **Yes**  **(n=26)** | **Odds Ratio (95% CI)** | **P-value** | | **Odds Ratio (95% CI)** | **P-value** | |
| Female (n=21) | 13 (56.5%) | 8 (30.8%) | 0.34 (0.11-1.10) | 0.073 | | 0.31 (0.06-1.64) | 0.170 | |
| Steroid Use (n=37) | 16 (69.6) | 21 (80.8) | 1.84 (0.49-6.87) | 0.366 | | 1.55 (0.31-7.65) | 0.593 | |
| Premorbid ACE/ARB (n=14) | 5 (21.7) | 9 (34.6) | 1.91 (0.53-6.84) | 0.323 | | 0.93 (0.16-5.52) | 0.938 | |
| SOFA, mean (SD) | 9.0 (2.7) | 10.3 (2.4) | 1.22 (0.96-1.56) | 0.103 | | 1.18 (0.89-1.57) | 0.252 | |
| CCI, median (IQR) | 5 (3-7) | 6 (3-10) | 1.10 (0.91-1.34) | 0.325 | | 1.21 (0.90-1.62) | 0.201 | |
| Lactate, median (IQR) | 3.3 (1.8-5.9) | 3.8 (1.9-7.4) | 0.99 (0.85-1.16) | 0.909 | | 0.96 (0.79-1.16) | 0.675 | |
| Age, mean (SD) | 66.2 (10.7) | 67.7 (13.6) | 1.01 (0.96-1.06) | 0.670 | | 1.00 (0.93-1.09) | 0.940 | |
| NE, median (IQR) | 0.38 (0.28-0.58) | 0.48 (0.33-0.83) | 5.04 (0.74-34.33) | 0.098 | | 5.72 (0.82-39.75) | 0.077 | |
| Ang II, n (%) | 9 (39.1) | 16 (61.5) | 2.49 (0.79-7.87) | 0.121 | | 1.08 (0.22-5.35) | 0.924 | |

Table S5 Legend: Characteristics associated with 30-day mortality in the entire cohort, and with each progressively increasing NE stratum, in dose increments of 0.1 mcg/kg/min. The univariate analysis shows the odds ratio of mortality for each covariate and the regression analysis includes all variables in the model. CI, confidence interval; ACE, angiotensin converting enzyme inhibitor therapy; ARB, angiotensin receptor blocker therapy; SOFA, sequential organ failure assessment; CCI, Charlson Comorbidity Index; NE, norepinephrine equivalents (norepinephrine + epinephrine + 2.5*vasopressin). Ang II, angiotensin II.

**Table S6: Mortality by type of shock after IPTW adjustment**

| **A** |  |  | **Before IPTW** | **After IPTW** |
| --- | --- | --- | --- | --- |
| Group | Ang II | Conventional Therapy | OR (95% CI), P-Value | OR (95% CI), P-Value |
| All Patients | 152/275 (55.3%) | 305/536 (56.9%) | 0.65 (0.45-0.95), 0.025 | 0.74 (0.55-0.99), 0.040 |
| High Output shock | 35/60 (58.3%) | 36/66 (54.6%) | 0.87 (0.32-2.37), 0.784 | 1.24 (0.61-2.52), 0.548 |
| Low Output shock | 16/25 (64.0%) | 10/24 (41.7%) | 1.08 (0.22-5.35), 0.924 | 2.45 (0.78-7.74), 0.126 |

Table S6 Legend: Mortality rates of the Ang II and Conventional therapy cohorts between high output shock groups. Usage of steroid and SOFA were dropped among patients with high shock while gender and SOFA were removed among those without high shock during IPTW.
